# A randomized, open-label study comparing low-dose clevudine plus adefovir combination therapy with clevudine monotherapy in naïve chronic hepatitis B patients

**DOI:** 10.1007/s12072-014-9537-5

**Published:** 2014-05-25

**Authors:** Won Young Tak, Jin Mo Yang, Byung Ik Kim, Soon Koo Baik, Gab Jin Cheon, Kwan Soo Byun, Do Young Kim, Byung Chul Yoo

**Affiliations:** 1 Department of Internal Medicine, Kyungpook National University Hospital, Daegu, Republic of Korea; 2 Department of Internal Medicine, The Catholic University Medical College St. Vincent’s Hospital, Suwon, Republic of Korea; 3 Division of Gastroenterology and Hepatology, Kangbook Samsung Hospital, Sungkyunkwan University School of Medicine, Seoul, Republic of Korea; 4 Department of Internal Medicine, Yonsei University Wonju College of Medicine, Wonju, Republic of Korea; 5 Department of Internal Medicine, GangNeung Asan Hospital, University of Ulsan College of Medicine, Gangneung, Republic of Korea; 6 Department of Internal Medicine, Korea University Guro Hospital, Seoul, Republic of Korea; 7 Department of Internal Medicine, Yonsei University College of Medicine, Seoul, Republic of Korea; 8 Department of Internal Medicine, Samsung Medical Center, Sungkyunkwan University School of Medicine, Seoul, Republic of Korea

**Keywords:** Adefovir, Clevudine, Combination therapy, Hepatitis B virus, Resistance, Viral kinetics

## Abstract

**Purpose:**

Clevudine 30 mg showed potent antiviral activity with a marked post-treatment antiviral effect. However, long-term treatment with clevudine monotherapy induced resistance and myopathy in some cases. The objective of this study is to evaluate the preliminary efficacy and safety of the combination of clevudine 20 mg and adefovir compared to clevudine monotherapy.

**Methods:**

Seventy-four patients were randomized to either a combination of clevudine 20 mg and adefovir or clevudine 20 or 30 mg and were treated for 2 years. The viral kinetics for 24 weeks, virological response [VR; hepatitis B virus (HBV) DNA less than 300 copies/ml], and the biochemical response [BR; normal alanine aminotransferase (ALT)] were assessed.

**Results:**

There was no difference in baseline characteristics among the three groups. Viral kinetics study showed no statistically significant difference among them during 24 weeks. The combination group showed 95 % virological response with a statistically significant difference compared to the clevudine 30 mg (67 %) and 20 mg (71 %) groups (*p* = 0.0376). Biochemical response rates were similar in all groups (78–94 %). No resistance was reported in the combination group, while 20 % of patients treated with clevudine 30 mg or 20 mg reported resistance during 2 years. Muscle-related symptoms such as myalgia (1 in clevudine 30 mg, 1 in the combination group) and muscle weakness (1 in clevudine 30 mg, 2 in clevudine 20 mg) were reported in five patients (7 %); of these, three patients discontinued the study.

**Conclusion:**

We concluded that the combination of clevudine 20 mg and adefovir produced a potent antiviral response together with a good resistance profile compared to clevudine monotherapy at 96 weeks in this pilot study.

## Introduction

Although effective vaccines are available in many countries, hepatitis B virus (HBV) infection still constitutes a global health threat since it can develop into liver cirrhosis and hepatocellular carcinoma [1–3].

There are several oral antiviral agents, including lamivudine, adefovir, telbivudine, entecavir, and tenofovir, available worldwide, and clevudine in Korea the and Philippines, for the treatment of HBV infection. These drugs were used as monotherapy in their clinical trials. However, drug-induced mutations often emerged during monotherapy with nucleos(t)ides, which were associated with viral breakthrough and clinical deterioration [4]. Recently, the combination of nucleos(t)ides has been recommended to avoid such mutations [5] despite its non-synergic effect [6].

Clevudine [7] showed very potent antiviral activity with the unique advantage of sustained viral suppression after withdrawal of treatment, which was demonstrated in several clinical studies [8–10]. However, long-term therapy showed the development of drug resistance [(0.7, 7.6 %) for 1 year] [11, 12] and skeletal myopathy [(1.7, 3.9 %) for 96 weeks] [12–15]. The clevudine-related mutation was rtM204I, as reported in previous publications [16]. Adefovir dipivoxil, which is an acyclic phosphonate, is not a highly potent drug against wild-type hepatitis B virus, but relatively potent against mutations with rtM204I [17–19].

In this study, we predicted that combination treatment with clevudine and adefovir may have additive or synergistic antiviral activity in patients with chronic hepatitis B because adefovir acts as a chain terminator and reduces the emergence of resistance.

Global studies on clevudine were voluntarily suspended in the USA by the sponsor because of the myopathy reported in Korea; clevudine had only been approved in Korea at that time. However, the Korean FDA scrutinized all of the safety data including muscle-related symptoms and then decided that clevudine could be marketed because its associated myopathy is not life threatening and is reversible when the patient is taken off the drug. Based on the results of Emax modeling using AAUCMB (HBV DNA average area under the curve minus baseline), the maximal predicted treatment efficacy of clevudine was 77, 91, and 94 % with doses of 10, 30, and 50 mg, QD, respectively [8].

We speculate that the reduced amount of clevudine at 20 mg can produce almost the same antiviral activity as clevudine 30 mg based on Emax modeling and may reduce the incidence of myopathy.

This study was designed to evaluate the preliminary antiviral activity and safety of the combination of clevudine 20 mg and adefovir versus monotherapy of clevudine 30 mg and 20 mg.

## Materials and methods

### Study design

This was a prospective, randomized, open-label trial to evaluate the preliminary efficacy and safety of the combination of clevudine 20 mg and adefovir compared to clevudine monotherapy in chronic hepatitis B patients enrolled at eight clinical centers in South Korea. All patients were randomly assigned to be treated with either clevudine 30 mg, 20 mg, or a combination of clevudine 20 mg and adefovir in a 1:1:1 ratio. The randomization list was produced using SAS before the study. Randomization was done with stratification on the basis of the study site, in blocks of three or six.

Patients were monitored at baseline, days 4, 7, 10, 14, weeks 3, 4, 8, 12, 24, 36, 48, 60, 72, 84, and 96 during the study period. Patients underwent physical examinations and blood samplings to measure laboratory parameters and HBV DNA levels according to the protocol. A viral kinetics study during the 24-week treatment period was also performed for all the enrolled patients.

The study was conducted in compliance with the principles of the Declaration of Helsinki and in accordance with Good Clinical Practice guidelines. Written informed consent, which was approved by the Institutional Review Board, was obtained from all of the subjects before they were examined for eligibility criteria. This study is registered as NIH clinical trial NCT01264354.

### Study population

Eligible patients were 18 years and older and had been hepatitis B surface antigen positive for at least 6 months. Patients had HBV DNA levels higher than 1 × 10^5^ copies/ml and abnormal ALT levels. Patients were asked to give written informed consent prior to study start and to comply with the study requirements. Patents who had been receiving interferon or peg-interferon within 6 months before enrollment were excluded. Patients previously treated with clevudine, lamivudine, adefovir, entecavir, telbivudine, tenofovir, or any other investigational drug for HBV infection were also excluded. The exclusion criteria included currently receiving antiviral, immunomodulatory, cytotoxic, or corticosteroid therapy; clinical evidence of decompensated liver disease such as total bilirubin ≥2.0 mg/dl; prothrombin time ≥1.7 (INR); albumin <3.5 g/dl; co-infection with hepatitis C, D, or the human immunodeficiency virus; evidence of ascites, variceal hemorrhage, or hepatic encephalopathy or hepatocellular carcinoma; history of liver transplantation. Patients who were pregnant or breast-feeding were also excluded.

### Efficacy endpoints

The primary endpoint was the proportion of patients with HBV DNA less than 300 copies/ml by real-time PCR at week 24. HBV DNA levels were measured at a central laboratory using the COBAS AmpliPrep/COBAS TaqMan HBV Test, v2.0 (Roche, Branchburg, NJ, USA), with a detection limit of 116 copies/ml.

Secondary endpoints included the reduction in HBV DNA, as defined as a mean log_10_ decrease from baseline, the proportion of patients with normal ALT, hepatitis B envelop antigen (HBeAg) loss, and/or seroconversion. The viral breakthrough was defined as a 1 log_10_ increase from nadir during the treatment period. RFMP analysis (Invitrogen, Carlsbad, CA, USA) on rtM204 and rtM180 sites was performed on the patients who showed viral breakthrough.

### Viral kinetics over the first 24 weeks

Serial HBV DNA samples were analyzed by a viral load function previously applied to the clearance kinetics of HBV from serum during a lamivudine and clevudine viral dynamics study [20]. The efficacy (*ε*) of inhibition of viral production, free virus clearance rate constant (*μ*), and infected cell loss rate constant (*a*) were determined by fitting the viral load function to the data using non-linear regression.

$$ V(t) = V_{0} e^{ - \mu \, t} + \frac{{(1 - \varepsilon )\mu V_{0} (e^{ - a \, t} - e^{ - \mu \, t} )}}{\mu - a} $$

### Safety analysis

Safety analysis included data from all 73 eligible patients who received at least one dose of study medication after randomization. Adverse events (AE), serious adverse events (SAE), and laboratory toxicity were included in the safety evaluations. If toxicities were not presented at baseline but appeared during the trial, or worsened in severity from baseline, laboratory toxicities were recorded. Muscle-related symptoms, including myopathy, were evaluated according to the guideline attached to the protocol.

### Statistical analysis

The results were analyzed on an intention-to-treat basis for the efficacy analysis. Patients discontinuing the study after receiving the first study drug dose were included in the efficacy analysis until their discontinuation. The patients who received at least one of the study medications after randomization were included in the safety analysis.

Statistical analysis was performed using SAS version 9.2 (SAS Institute, Cary, NC, USA). Determination of statistical significance was performed with an alpha level of 0.05. Comparisons of categorical variables were performed by chi-square test or Fisher’s exact test, and continuous variables were analyzed by one-way ANOVA test or Kruskal–Wallis test.

## Results

### Study population

A total of 73 eligible patients were enrolled at 8 sites and randomly assigned to receive the clevudine 30 mg daily (*n* = 25), clevudine 20 mg daily (*n* = 24), or the combination (clevudine 20 mg and adefovir) daily (*n* = 24). Baseline characteristics including the HBV DNA level and ALT levels were similar among the three groups (Table 1). At baseline, HBeAg-positive patients were 72 % (*N* = 18), 63 % (*N* = 15), and 63 % (*N* = 15) in the clevudine 30 mg, 20 mg, and combination group, respectively.

**Table 1:** Baseline characteristics

Characteristics	CLV 30 mg (*n* = 25)	CLV 20 mg (*n* = 24)	CLV 20 mg + ADV 10 mg (*n* = 24)	*p* value
Male (%)	52.00	62.50	50.00	0.6451^a^
Age (year)	44.16 ± 10.14	45.08 ± 12.83	48.92 ± 11.73	0.3233^b^
Weight (kg)	64.66 ± 12.00	64.07 ± 11.05	64.65 ± 12.71	0.9806^b^
HBV DNA (log copies/ml)^d^	7.21 ± 1.41	7.47 ± 1.04	7.31 ± 1.45	0.5257^c^
ALT (U/l)	73.96 ± 62.02	132.63 ± 145.13	125.79 ± 162.46	0.2437^c^
HBsAg (log IU/ml)^e^	3.53 ± 0.78	3.66 ± 0.68	3.64 ± 0.68	0.8302^b^
HBeAg positive (%)	72.00	62.50	62.50	0.7193^a^
LC (%)	32.00	25.00	20.83	0.6661^a^

Data are expressed as mean ± SD (standard deviation)

^a^Chi-squre test

^b^ANOVA test

^c^Kruskal–Wallis test

^d^013-R007 (CLV 20 mg) was excluded because of missing data

^e^57 patients (CLV 30 mg: 18, CLV 20 mg: 17, CLV 20 mg + ADV 10 mg: 22) who completed the week 96 visit

A total of 16 patients withdrew from the study. Hence, 57 patients (18 in the clevudine 30 mg group, 17 in the clevudine 20 mg group, and 22 in the combination group) completed the 96-week treatment period. Seven patients discontinued the study in the clevudine 30 mg group because of resistance (4), adverse events (2), and loss to follow-up (1); 7 patients in the clevudine 20 mg group because of resistance (3), withdrawal of consent (2), adverse events (1), and loss to follow-up (1); 2 patients in the combination group because of withdrawal of consent (2). For the viral kinetics analysis, 24, 23, and 20 patients were included in the clevudine 30 mg, 20 mg, and combination groups, respectively.

### Virologic and serologic endpoints

The proportions of patients with HBV DNA levels less than 300 copies/ml by real-time PCR assay at 24 weeks, which was a primary efficacy endpoint, were 60, 59, and 57 % in the clevudine 30 mg, 20 mg, and combination groups, respectively, without a statistically significant difference (*p* = 0.9688) (Table 2). The proportions of patients with HBV DNA levels less than 300 copies/ml at week 96 were 67, 71, and 95 % in the clevudine 30 mg, 20 mg, and combination groups, respectively, which showed statistically significant differences (*p* = 0.0376).

**Table 2:** Comparison of the virologic and biochemical response rates

	CLV 30 mg	CLV 20 mg	CLV 20 mg + ADV 10 mg	*p* value
Virologic response (<300 copies/ml)				
Week 24	60.00 % (15/25)	59.09 % (13/22)	56.52 % (13/23)	0.9688^a^
Week 48	66.67 % (16/24)	70.00 % (14/20)	73.91 % (17/23)	0.8629^a^
Week 72	63.64 % (14/22)	66.67 % (12/18)	81.82 % (18/22)	0.3692^a^
Week 96	66.67 % (12/18)	70.59 % (12/17)	95.45 % (21/22)	0.0376^b^
Biochemical response (normal ALT)				
Week 24	76.00 % (19/25)	81.82 % (18/22)	69.57 % (16/23)	0.6313^a^
Week 48	91.67 % (22/24)	85.00 % (17/20)	82.61 % (19/23)	0.6695^b^
Week 72	81.82 % (18/22)	88.89 % (16/18)	81.82 % (18/22)	0.8290^b^
Week 96	77.78 % (14/18)	94.12 % (16/17)	86.36 % (19/22)	0.4053^b^

^a^Chi-square test

^b^Fisher’s exact test

**Fig. 1:**
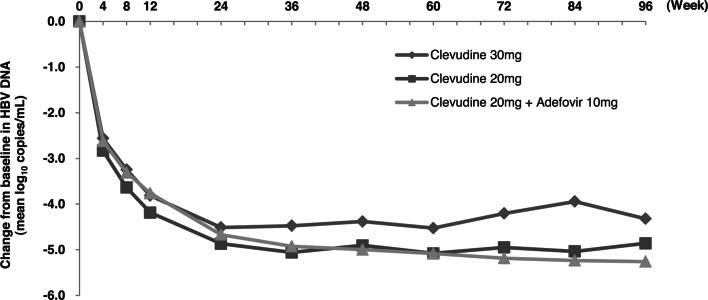
Mean changes from baseline in HBV DNA. Mean HBV DNA changes from baseline at week 96 were −4.32, −4.86, and −5.26 log_10_ copies/ml in the clevudine 30 mg, clevudine 20 mg, and combination groups, respectively, which did not show a statistically significant difference (*p* < 0.3534)

The mean HBV DNA changes from baseline at week 96 were −4.32 ± 1.63, −4.86 ± 1.24, and −5.26 ± 1.42 log_10_ copies/ml in the clevudine 30 mg, 20 mg, and combination groups, respectively, which did not show statistically significant differences (*p* < 0.3534) (Fig. 1). At week 96, the rates of HBeAg loss (21 % in the clevudine 30 mg, 40 % in the clevudine 20 mg, and 21 % in the combination group) and/or HBeAg seroconversion (7 % in the clevudine 30 mg, 40 % in the clevudine 20 mg, and 21 % in the combination group) were comparable among the three groups (*p* = 0.5809, 0.1684). Viral breakthroughs were observed in 28 % of the clevudine 30 mg, 17 % of the clevudine 20 mg, and 0 % of the combination group, which was significantly different (*p* = 0.0149). Among them, six patients in the clevudine 30 mg and five patients in the clevudine 20 mg group showed genotypic mutation at rt204I; three of these patients showed double mutations at rtM204I and rtL180M. In conclusion, clevudine-related mutation was reported in 20 % of the clevudine monotherapy group, while no viral breakthrough was observed in the combination group.

### Viral kinetics over the first 24 weeks

Viral dynamics over the first 24 weeks were analyzed for 67 subjects who completed treatment at 24 weeks. Table 3 summarizes the estimated parameters by treatment group. The* p* values of the Kruskal–Wallis test were 0.934, 0.489, and 0.173 for $\varepsilon$, $\mu$, and $\alpha$, respectively, which did not show statistically significant differences in the median estimates among the three groups.

**Table 3:** Viral dynamic over 24 weeks

Parameter	Group	*N* ^b^	N Miss^c^	Mean	SD	Median	Min.	Max.	*p* value^a^
$\varepsilon$	CLV 30 mg	24	1	0.877	0.201	0.959	0.120	1.000	0.934
	CLV 20 mg	23	0	0.927	0.085	0.957	0.653	0.994	
	CLV + ADV	20	3	0.911	0.095	0.959	0.703	1.000	
$\mu$	CLV 30 mg	24	1	1.672	2.000	0.761	0.150	6.331	0.489
	CLV 20 mg	23	0	2.026	2.199	1.063	0.304	6.419	
	CLV + ADV	20	3	1.817	1.927	0.826	0.261	6.083	
$\alpha$	CLV 30 mg	24	1	0.139	0.123	0.121	0.003	0.495	0.173
	CLV 20 mg	23	0	0.166	0.083	0.130	0.056	0.337	
	CLV + ADV	20	3	0.127	0.099	0.078	0.003	0.320	

^a^Kruskal–Wallis test

^b^Patients (CLV 30 mg: 013-R010, CLV 20 mg + ADV 10 mg: 013-R006, 062-R004, 062-R008) were excluded because of numerical issues (estimates were biased)

^c^Patients (CLV 20 mg: 013-R007, CLV 20 mg + ADV 10 mg: 022-R001) were excluded because of dropping out before 24 weeks

### Biochemical endpoints

The proportions of patients who had normal ALT at week 96 were 78, 94, and 86 % in the clevudine 30 mg, 20 mg, and combination groups, respectively (*p* < 0.4053) (Table 2).

### Safety and tolerability

During the 96-week treatment period, the incidences of adverse events were similar among the three groups: 68, 63, and 58 % in the clevudine 30 mg, 20 mg, and combination groups, respectively (*p* < 0.7808). The most frequent adverse events, occurring in more than 10 % of patients, were hypertension (20 %) and CPK elevation (12 %) in the clevudine 30 mg group and upper respiratory tract infection (17 %) and CPK elevation (13 %) in the clevudine 20 mg group. No event occurred in more than 10 % of patients in the combination group. The incidence of serious adverse events during treatment was 12 % (3 patients) only in the clevudine 30 mg group. The SAEs of angina pectoris, arrhythmia, and inguinal hernia reported by three patients were mild or moderate in severity and considered to be unrelated to the study drugs.

Muscle-related symptoms such as myalgia and muscle weakness were reported in two patients (1 of myalgia, 1 of muscle weakness) in the clevudine 30 mg group, two patients (muscle weakness) in the clevudine 20 mg group, and one patient (myalgia) in the combination group.

Laboratory toxicities at grade 3 or higher were reported in eight patients, three patients, and four patients in the clevudine 30 mg, 20 mg, and combination groups, respectively, and the difference was not statistically significant (*p* = 0.2738).

The CPK levels at grade 3 or higher were reported in 4, 1, and 3 patients in the clevudine 30 mg, 20 mg, and combination groups, respectively, during the study. Two patients out of eight with CPK elevation of more than grade 3 showed muscle-related symptoms in the clevudine 30 mg group and discontinued clevudine therapy. The other six patients continued therapy, and their CPK levels stabilized.

## Discussion

The baseline characteristics of the three groups were well balanced in this randomized study.

Based on the viral dynamics study over 24 weeks of treatment, it was speculated that the antiviral activity of the combination of clevudine 20 mg and adefovir 10 mg was as good as that of clevudine 30 mg.

Clevudine is known to have highly potent antiviral activity. However, the emergence of resistance during therapy is a limitation to long-term treatment. The combination of clevudine 20 mg and adefovir also provided very potent antiviral activity, although the dose of clevudine was reduced from 30 to 20 mg.

The proportion of patients with HBV DNA less than 300 copies/ml by real-time PCR at week 96 was higher in the combination group (95 %) compared to the clevudine monotherapy groups (67, 71 %) (*p* < 0.0376), which resulted from the viral breakthrough led by the emergence of resistance in the clevudine monotherapy groups. Although direct head-to-head comparisons are not available, the antiviral activity of the combination was similar to that of tenofovir (76 % HBeAg-positive and 93 % HBeAg-negative patients with HBV DNA less than 400 copies/ml at week 48) [21]. We also evaluated the proportion of patients with HBV DNA less than 116 copies/ml by real-time PCR: 86 % in the combination of clevudine 20 mg and adefovir group. This result demonstrated remarkable viral suppression activity by the combination therapy. For the patients with liver cirrhosis at baseline, the proportions of patients with HBV DNA levels less than 300 copies/ml did not show statistically significant differences among the three groups at week 96 (*p* = 1.000, data not shown).

In this study, it was demonstrated that combination therapy with clevudine 20 mg and adefovir did not induce resistance, while 20 % of resistance was reported in the clevudine monotherapy groups. Clevudine is a nucleoside analog and shows cross resistance to lamivudine and telbivudine, which belong to the same group of nucleoside analogs. In contrast, adefovir is a nucleotide analog. Therefore, we can speculate that combination therapy with different structures would have an advantage over monotherapy on emergence of resistance.

The biochemical response, which was defined as the proportion of normal ALT at week 96, was similar among the three groups (*p* = 0.4053). We had a limitation in the evaluation of serological response because 28–38 % of HBeAg-negative patients were included in each group, including a small number of patients.

Also, we investigated the hepatitis B surface antigen (HBsAg) reduction in 57 patients who completed the week 96 visit. The mean declines of HBsAg level from baseline were −0.03 ± 0.53, −0.18 ± 0.32, and −0.25 ± 0.47 log IU/ml in the clevudine 30 mg, 20 mg, and combination groups, respectively. In our study, HBsAg reduction was not obvious, although HBsAg reduction by clevudine has been published in previous papers [22, 23]. In consideration of the low level of baseline HBsAg (3.53–3.66 log IU/ml) in this study, we can presume that HBsAg reduction by clevudine is more predominant in patients with high baseline HBsAg levels.

Adefovir dipivoxil is known to develop nephrotoxicity with serum creatinine elevation during administration [24]. Therefore, we investigated the change in serum creatinine in the combination groups, which did not show a statistically significant difference at week 96 from baseline (data not shown). No patients showed symptoms of nephrotoxicity during the treatment period.

During the 2-year treatment period, muscle weakness was reported in three patients treated with clevudine only. Two patients with muscle weakness in the clevudine 20 mg group continued with the study, and the symptom disappeared during clevudine treatment. One patient with muscle weakness in the clevudine 30 mg group discontinued with the study. The symptom of this patient was resolved after stopping clevudine treatment. Two patients with myalgia reported (1 in the clevudine 30 mg and 1 in the combination group) continued with the study, and their symptoms were resolved during the study.

We planned to investigate whether the dose reduction of clevudine from 30 to 20 mg would affect the incident rate of myopathy by clevudine. Considering these data, it is hard to conclude that the dose of clevudine is connected to muscle-related symptoms. In our study, there was no relationship between muscle weakness and CPK elevation, while it seemed that muscle weakness was followed by CPK elevation.

Due to the limitation of the sample size, a large-scale clinical study is required for the evaluation of the relationship between muscle-related symptoms and the dose, as well as the efficacy of combination therapy.
